# External validation of risk classification in patients with docetaxel-treated castration-resistant prostate cancer

**DOI:** 10.1186/1471-2490-14-31

**Published:** 2014-04-18

**Authors:** Kazuhiko Nakano, Kenji Komatsu, Taro Kubo, Shinsuke Natsui, Akinori Nukui, Shinsuke Kurokawa, Minoru Kobayashi, Tatsuo Morita

**Affiliations:** 1 Department of Urology, Jichi Medical University, Yakushiji 3311-1, Shimotsuke, Tochigi 329-0498, Japan

**Keywords:** Castration-resistant prostate cancer, Docetaxel, Risk classification, Validation study

## Abstract

**Background:**

Castration-resistant prostate cancer (CRPC) patients have poor prognoses, and docetaxel (DTX) is among the few treatment options. An accurate risk classification to identify CRPC patient groups for which DTX would be effective is urgently warranted. The Armstrong risk classification (ARC), which classifies CRPC patients into 3 groups, is superior; however, its usefulness remains unclear, and further external validation is required before clinical use. This study aimed to examine the clinical significance of the ARC through external validation in DTX-treated Japanese CRPC patients.

**Methods:**

CRPC patients who received 2 or more DTX cycles were selected for this study. Patients were classified into good-, intermediate-, and poor-risk groups according to the ARC. Prostate-specific antigen (PSA) responses and overall survival (OS) were calculated and compared between the risk groups. A multivariate analysis was performed to clarify the relationship between the ARC and major patient characteristics.

**Results:**

Seventy-eight CRPC patients met the inclusion criteria. Median PSA levels at DTX initiation was 20 ng/mL. Good-, intermediate-, and poor-risk groups comprised 51 (65%), 17 (22%), and 10 (13%) patients, respectively. PSA response rates ≥30% and ≥50% were 33%, 41%, and 30%, and 18%, 41%, and 20% in the good-, intermediate-, and poor-risk groups, respectivcixely, with no significant differences (p = 0.133 and 0.797, respectively). The median OS in the good-, intermediate-, and poor-risk groups were statistically significant (p < 0.001) at 30.1, 14.2, and 5.7 months, respectively. A multivariate analysis revealed that the ARC and PSA doubling time were independent prognostic factors.

**Conclusions:**

Most of CRPC patients were classified into good-risk group according to the ARC and the ARC could predict prognosis in DTX-treated CRPC patients.

**Trial registration:**

University Hospital Medical Information Network Clinical Trials Registry (UMIN-CTR) number, UMIN000011969.

## Background

Castration-resistant prostate cancer (CRPC) patients have poor prognoses. Although many treatment options have been developed, truly effective ones remain limited [1-6]. In Japan, the currently available drugs are limited even further. Predictions and classifications of CRPC patients’ clinical outcomes and prognoses for the effective use of the limited treatment options offer prolonged survival to the patients. In particular, docetaxel (DTX) [1,2] has been established as effective and has become widely used in CRPC treatment; however, in some patients, DTX is ineffective and induces a high incidence of adverse events. Thus, the development of an accurate risk classification that can identify the CRPC patient group in which DTX would be effective is urgently warranted. Although some reports have demonstrated the usefulness of superior nomograms for predicting prognosis in CRPC patients [7-9], these nomograms include many investigation items and are therefore somewhat difficult to implement in clinical practice. The Armstrong risk classification (ARC), which classifies CRPC patients into 3 groups according to 4 risk factors, including visceral metastases, bone scan progression, significant pain, and anemia (hemoglobin [Hb] level < 13 g/dL), is also a superior risk classification because it can be easily used in clinical practice without reducing the predictive abilities of nomograms and can predict not only survival but also post-chemotherapy prostate-specific antigen (PSA) declines and tumor responses [10]. ARC is highly reliable because it was developed from 656 CRPC patients who were administered DTX and was also internally validated in 333 CRPC patients who were administered mitoxantrone among the 1006 CRPC patients in the TAX327 study [1]. Furthermore, ARC was demonstrated to significantly classify the clinical outcomes of estramustine phosphate (EMP) treatment in CRPC patients [11].

However, few reports have externally validated ARC in CRPC patients who were administered DTX. Under external validation, risk classifications and nomograms might be found to have positive [12] or negative [13] effects and, sometimes, to clarify characteristics at the time of clinical use [14]. Kawahara et al. [15] reported that CRPC patients who were administered DTX in 10 or more cycles had favorable prognosis; in this study, the authors examined whether ARC would be useful when selecting CRPC patients who could continue a DTX regimen for 10 or more cycles. However, the Hb criteria were changed to 10 g/dL from 13 g/dL, the bone scan progression risk factor was replaced with alkaline phosphatase (ALP) levels, and the association between PSA response and ARC was not referenced. Armstrong, the developer of the ARC, externally validated ARC and the above-mentioned nomograms in CRPC patients who were administered DTX [16] and indicated the superior but insufficient discriminatory abilities and necessary improvements of these tools. Thus, the usefulness of ARC remains unclear and needs further external validation before clinical use.

The objective of this study was to examine the clinical significance of ARC through external validation in DTX-treated Japanese CRPC patients.

## Methods

### Patients and treatment

This study was approved by the institutional review board of Jichi Medical University. The clinical trial was registered in the University Hospital Medical Information Network Clinical Trials Registry (UMIN-CTR) UMIN000011969. Written informed consent to participate in this study was obtained from all patients. At our institution, patients with metastatic and/or first treatment-refractory prostate cancer (PCa) are treated with androgen deprivation therapy (ADT). After progressing to CRPC, the patients are principally treated in the following order: 1) combined androgen blockade (CAB), 2) anti-androgen withdrawal, 3) anti-androgen substitution, 4) EMP, 5) DTX, 6) dexamethasone, and 7) best supportive care. These treatments are continued until disease progression and/or unacceptable toxicity occurs. Of the CRPC patients who received DTX in our institution between July 2003 and September 2012, those who met the following inclusion criteria were eligible for this study: 1) confirmed histological PCa diagnosis, 2) refractory to ADT with CAB, anti-androgen withdrawal, and anti-androgen substitution, 3) refractory to EMP, and 4) received 2 or more cycles of DTX.

A modified version of the regimen used in the SWOG9916 study [2] was used as the DTX treatment protocol [17]. Briefly, DTX (60 mg/m^2^) was administered by intravenous drip infusion for 1 hour on day 1, once every 3–4 weeks. Twice-daily EMP (280 mg) was orally administered in combination with DTX. EMP could be reduced to 280 mg/day according to the degree of adverse events and, if already administered before DTX initiation, continued at the same dose that was administered before DTX treatment. As a premedication, 8 mg of dexamethasone was administered by intravenous drip infusion before and after the DTX treatment. The DTX treatment was continued until disease progression, unacceptable toxicity, or a patient’s request for its cessation. Disease progression was defined as increases in the number of evaluable lesions observed on imaging tests and/or biological progression characterised by an elevated serum PSA level of 25% and an absolute increase of 2 ng/mL or higher than the nadir in at least 3 consecutive measurements.

### Armstrong risk classification

The patients were classified as good-, intermediate-, and poor-risk according to the ARC, which included the following 4 risk factors: visceral metastases, bone scan progression, significant pain, and anemia (Hb level < 13 g/dL) [10]. Patients with 0 or 1, 2, and 3 or 4 risk factors were classified as good-, intermediate-, and poor-risk, respectively. The risk factor of visceral metastases was defined as “presence” if computed tomography (CT) and/or magnetic resonance imaging (MRI) were performed at DTX initiation and revealed visible visceral metastases. The risk factor of bone scan progression was subject to satisfaction that a bone scan had been performed at DTX initiation, comparable prior bone scans had also been performed, and progression or increases in the numbers of hot spots were demonstrated by these scans. Although significant pain was defined as a Present Pain Intensity score (PPI) ≥ 2 and/or an analgesic score (AS) ≥ 10 in the ARC [10], we defined the use of some types of analgesic at DTX initiation as a surrogate measurement of significant pain because the PPI and AS of the patients in this study were not measured. The risk factor of anemia was defined as “presence” if the patient met the criteria of Hb levels < 13 g/dL at DTX initiation.

### Assessment

According to the recommendations of the Prostate Cancer Clinical Trial Working Group [18], PSA responses were demonstrated in waterfall plot of decreasing PSA rates for each patient. Decreasing PSA rates were obtained from the values determined just before DTX initiation and the lowest PSA values during DTX treatment. Overall survival (OS) was defined as the period from DTX initiation to death. When patients were lost to follow-up, OS was considered up to the last day of confirmed patient survival. Adverse events were determined according to the National Cancer Institute Common Toxicity Criteria (NCI-CTC) version 3.

### Statistical analysis

PSA responses were compared with a chi-square test. OS was determined according to the Kaplan-Meier method and compared with the log-rank test. A multivariate analysis of OS was performed to compare the prognostic factors in a Cox proportional hazard analysis. Continuous data were divided into 2 groups according to median value. A concordance index (c-index) was estimated as a measure of the ARC discriminatory index. A c-index of 0.50 represents random prediction, whereas a c-index of 1.0 represents a perfect discriminatory ability [9,10,19]. P < 0.05 was considered statistically significant.

## Results

### Patients

During the study period, 102 CRPC patients received DTX at our institution, among whom, 78 met the inclusion criteria. The patient characteristics are shown in Table 1. The median observation period was 24 months (range, 3–74 months). The median number of administered DTX cycles was 5 (range, 2–46 cycles), and the median DTX administration period was 9 months (range, 1–66 months). In addition, 0, 1, 2, 3, and 4 ARC risk factors were observed in 9, 42, 17, 9, and 1 patients, respectively. The good-, intermediate-, and poor-risk groups according to the ARC included 51 (65%), 17 (22%), and 10 (13%) patients, respectively. CRPC patients with a history of EMP use were 47/51 (92%), 14/17 (82%), and 8/10 (80%) patients in the good-, intermediate-, and poor-risk groups, respectively, with no statistically significant difference (p = 0.367). A total of 67 patients (86%) discontinued DTX treatment during the observation period, of whom 51, 8, 2, 4, and 2 patients discontinued DTX treatment because of disease progression, adverse events, death, patient request, and other reasons, respectively. The remaining 11 patients (14%) were still undergoing DTX treatment during the course of the study.

**Table 1:** Patient characteristics

		**(n = 78)**
Age (years)		70 (50–88)
PSA at PCa diagnosis (ng/mL)		124.6 (4.7-19523.1)
PSA at DTX initiation (ng/mL)		19.7 (0.6-1053.0)
Time from PCa diagnosis to DTX initiation (months)		37 (4–189)
PSADT (months)		2.4 (0.6-33.9)
ECOG performance status, n (%)		
0		40 (51)
1		28 (36)
2		10 (13)
Gleason score, n (%)		
<6		5 (6)
7		16 (21)
>8		50 (64)
Unknown		7 (9)
Metastatic site, n (%)		
Bone		42 (54)
Lymph nodes		19 (24)
Liver		3 (4)
Lung		1 (1)
None		28 (36)
Bone scan progression, n (%)		
Yes		15 (19)
No		63 (81)
Pain at baseline, n (%)		
Yes		24 (31)
No		54 (69)
Haemoglobin (g/dL)		11.8 (8.4-14.1)
ALP (IU/L)		290 (59–8689)
Prior treatment, n (%)		
Combined androgen blockade		78 (100)
Prostatectomy		3 (4)
Radiotherapy		10 (13)
Estramustine		69 (88)
No. of DTX cycles		5 (2–46)

*Abbreviations:* PSA, prostate-specific antigen; PCa, prostate cancer; DTX, docetaxel; PSADT, prostate-specific antigen doubling time; ECOG, Eastern Cooperative Oncology Group; ALP, alkaline phosphatase. All continuous data are described in median (range).

### Armstrong risk classification assessment

**Figure 1:**
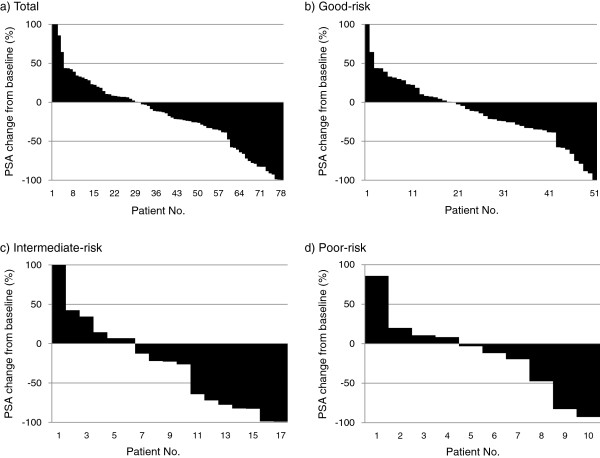
Waterfall plots of prostate-specific antigen (PSA) responses according to total (a), good-(b), intermediate-(c), and poor-(d) risk group of Armstrong risk classification.

Waterfall plots of PSA response according to each ARC risk group are shown in Figure 1. PSA responses ≥0%, ≥30%, and ≥50% were observed in 48 (62%), 27 (35%), and 18 (23%) of the total patients, respectively. PSA response rates ≥30% and ≥50% were observed in 33%, 41%, and 30%, and 18%, 41%, and 20% of the good-, intermediate-, and poor-risk groups, respectively, with no statistically significant differences between the groups (p = 0.133 and 0.797, respectively).

The OS according to each ARC risk factor is shown in Table 2. Regarding the risk factor of anemia, which was divided into 2 groups according to the relatively high Hb value of 13 g/dL, the group with Hb levels ≤ 13 g/dL dominated with 64 patients (82%). There were significant associations between OS and the risk factors of visceral metastases (p < 0.001), bone scan progression (p < 0.001), and significant pain (p < 0.001), but not anemia (p = 0.442).

**Table 2:** **Univariate analysis for overall survival according to each risk factor of Armstrong risk classification **[10]

**Risk factor**	**Category**	**n**	**Median (months)**	**Hazard ratio**	**95% CI**	p value*
Visceral metastases	no	74	20.3	1.00	5.51 - 68.83	<0.001
	yes	4	3.9	19.47		
Bone scan progression	no	63	25.8	1.00	2.00 - 7.14	<0.001
	yes	15	9.1	3.78		
Significant pain	no	54	25.8	1.00	1.72 - 5.17	<0.001
	yes	24	8.7	2.98		
Hemoglobin (g/dL)	>13.0	14	22.9	1.00	0.66 - 2.60	0.442
	≦ 13.0	64	19.5	1.31		

*Abbreviations:* CI, confidence interval. *log rank test.

**Figure 2:**
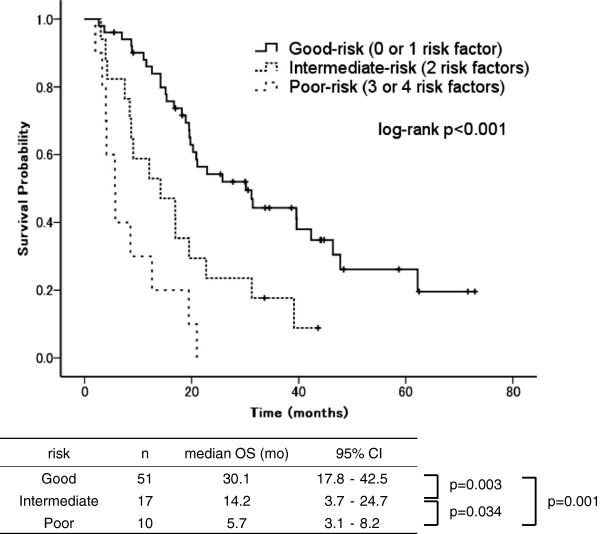
**Overall survival curves according to Armstrong risk classification **[10]**.**

Patient distributions and OS curves according to the ARC risk groups are shown in Figure 2. The CRPC patients at our institution were mostly classified as good-risk (65%). The median OS durations in the good-, intermediate-, and poor-risk groups were 30.1 months (95% confidence interval [CI]: 17.8–42.5 months), 14.2 months (95% CI: 3.7–24.7 months), and 5.7 months (95% CI: 3.1–8.2 months), respectively, with statistically significant differences between the groups (p < 0.001). During the observation period, death occurred in 39 patients (46%), of whom 18, 14, and 7 were in the good-, intermediate-, and poor-risk groups, respectively. The c-index was 0.60 for OS, indicating that the ARC had a modest discriminatory ability in our cohort.

### Subgroup analysis

A multivariate analysis was performed to clarify the relationship between the ARC and the major patient characteristics that are often evaluated in clinical practice. The following 6 factors at DTX initiation were covariates of interest: age, PSA level, PSA doubling time (PSADT), Eastern Cooperative Oncology Group (ECOG) performance status, Gleason score, and ARC. A univariate analysis conducted with the log-rank test revealed significant associations between OS and 4 factors (PSA at DTX initiation, PSADT, ECOG performance status, and ARC). A multivariate analysis with these 4 factors revealed that the ARC and PSADT were independent prognostic factors (Table 3).

**Table 3:** Univariate and multivariate analysis for overall survival of major prognostic factors

**Prognostic factor**	**Category**	**n**	**Median (months)**	**Univariate**			**Multivariate**		
				**Hazard ratio**	**95% CI**	p value*	**Hazard ratio**	**95% CI**	p value**
Age (years)	≦ 70	43	17.0	1.00	0.35 - 1.03	0.062			
	>70	35	22.7	0.60					
PSA at DTX initiation (ng/ml)	≦ 20	39	39.6	1.00	1.94 - 6.11	<0.001	1.00		0.058
	>20	39	12.6	3.44			2.07	0.98 - 4.37	
PSADT (months)	>2.4	38	31.4	1.00	1.48 - 4.41	0.001	1.00		0.033
	≦ 2.4	40	16.7	2.56			1.88	1.05 - 3.36	
ECOG performance status	≦ 1	68	20.3	1.00	1.55 - 6.26	0.001	1.00		0.740
	2	10	4.1	3.11			1.16	0.49 - 2.74	
Gleason score	≦ 7	21	31.2	1.00	0.89 - 3.00	0.113			
	>8	57	18.2	1.63					
Armstrong risk classification	Good	51	30.1	1.00			1.00		0.060
	Intermediate	17	14.2	2.48	1.32 - 4.66	<0.001	1.33	0.60 - 2.97	0.487
	Poor	10	5.7	6.58	3.07 - 14.11		3.21	1.17 - 8.80	0.024

*Abbreviations:* CI, confidence interval; PSA, prostate-specific antigen; PSADT, prostate-specific antigen doubling time; ECOG, Eastern Cooperative Oncology Group; ALP, alkaline phosphatase. *log rank test. **Cox proportional hazards model.

## Discussion

We externally validated the ARC in CRPC patients who were administered DTX in 2 or more cycles and showed that there were statistically significant differences in OS among the ARC risk groups. The median OS and 95% CI for each ARC risk group were similar between the CRPC patients used for validation in this study (the validation group) and the CRPC patients used to develop the ARC [10] (the development group). The c-index for OS was 0.60, indicating that the ARC had a modest discriminatory ability in the validation group. A multivariate analysis revealed that the ARC was an independent prognostic factor. Thus, the ability of the ARC to classify and approximately predict the OS of CRPC patients with certain reproducibility was confirmed, suggesting that the ARC is useful when predicting prognosis in DTX-treated CRPC patients. This means that CRPC patients who are classified into good- and intermediate-risk groups are recommended for aggressive DTX administration, because these patients would be expected to experience prolonged OS in response to DTX. However, CRPC patients who are classified as poor-risk should be recommended for clinical trial participation or other treatments because given the poor outcomes, these patients would be expected to experience a limited prognosis despite the use of DTX.

ARC was also reported to be able to classify PSA response in CRPC patients, although this system was principally aimed at classifying OS [10]. We also externally validated the usefulness of ARC in classifying the PSA response of CRPC patients. However, there were no statistically significant differences in PSA response between the ARC risk groups. This result was considered to be caused by the reason that most of CRPC patients in the validation group had a history of EMP use; the frequency of a history of EMP use was high in good-risk group (92%) compared to intermediate-risk (82%) and poor-risk (80%) groups. The CRPC patients with a history of EMP use showed significantly lower PSA response during DTX treatment than those without a history of EMP use [10]. Thus, considering that PSA response during DTX treatment are likely to be low in the CRPC patients with a history of EMP use, our cohort might not be suitable for validating the usefulness of ARC in classifying the PSA response of CRPC patients.

This validation study exhibited the following characteristics with respect to the ARC: the development group presented with median PSA levels of 110 ng/mL at DTX initiation. From this group, symptoms and imaging test items that are often observed after some disease progression, including bone scan progression [9], significant pain [20], and visceral metastases [8,21], were identified as risk factors. However, as the efficacy of DTX was established in CRPC patients and DTX initiation in patients with low PSA levels was found to confer better prognosis [9,15,17,21], the likelihood of initiating DTX at lower PSA levels has increased to a level higher than those reported in the TAX327 and SWOG9916 trials. The validation group presented median PSA levels of 20 ng/mL at DTX initiation, a lower value than that of the development group, and possessed few ARC risk factors; this led to a disproportionate distribution in which 65% of CRPC patients in the validation group were classified as good-risk. Thus, the ARC tends to classify many CRPC patients with low PSA levels as good-risk. At the comparison of the prognoses and/or treatment responses of CRPC patients, the ARC would ensure more accurate outcomes while considering the above-mentioned ARC characteristics.

This study had the following limitations: a retrospective study design; a small sample size; different patient backgrounds in the validation and development groups in terms of EMP exposure, lower PSA levels, lack of visceral spread, and lower numbers of DTX cycles; and a different definition of significant pain as a risk factor. Although these major limitations could have possibly deteriorated the quality of this external validation study of ARC, it was noteworthy that the ARC indicated good discriminatory ability for OS even in this validation group.

## Conclusions

Most of CRPC patients were classified into good-risk group according to the ARC and the ARC could predict prognosis in DTX-treated CRPC patients.

## Competing interests

The authors declare that they have no competing interests.

## Authors’ contributions

This study has been designed by KN and TM. The clinical database of the patients have been acquired by KN, KK, TK, SN, AN, SK, MK and TM. Manuscript has been written by KN and TM. KN is responsible for the statistical analyses. Conclusions have been drawn mainly by KN and TM. TM has given final approval of the version to be published. All authors read and approved the final manuscript.

## Pre-publication history

The pre-publication history for this paper can be accessed here:

http://www.biomedcentral.com/1471-2490/14/31/prepub
